# Differential Roles of Each Orexin Receptor Signaling in Obesity

**DOI:** 10.1016/j.isci.2019.09.003

**Published:** 2019-09-09

**Authors:** Miyo Kakizaki, Yousuke Tsuneoka, Kenkichi Takase, Staci J. Kim, Jinhwan Choi, Aya Ikkyu, Manabu Abe, Kenji Sakimura, Masashi Yanagisawa, Hiromasa Funato

**Affiliations:** 1 International Institute for Integrative Sleep Medicine (WPI-IIIS), University of Tsukuba, Tsukuba, Ibaraki 305-8575, Japan; 2 Department of Anatomy, Faculty of Medicine, Toho University, Ota-ku, Tokyo 143-8540, Japan; 3 Laboratory of Psychology, Jichi Medical University, Shimotsuke, Tochigi 329-0498, Japan; 4 Department of Cellular Neurobiology, Brain Research Institute, Niigata University, Niigata 951-8585, Japan; 5 Department of Animal Model Development, Brain Research Institute, Niigata University, Niigata 951-8585, Japan; 6 Tsukuba Advanced Research Alliance (TARA), University of Tsukuba, Tsukuba, 305-8575 Ibaraki, Japan; 7 Department of Molecular Genetics, University of Texas Southwestern Medical Center, Dallas, TX 75390, USA

**Keywords:** Physiology, Cellular Physiology, Endocrinology, Diabetology

## Abstract

Orexins are hypothalamic neuropeptides that regulate feeding, energy expenditure, and sleep. Although *orexin*-deficient mice are susceptible to obesity, little is known about the roles of the orexin receptors in long-term energy metabolism. Here, we performed the metabolic characterization of orexin receptor-deficient mice. *Ox1r*-deficient mice were resistant to diet-induced obesity, and their food intake was similar between chow and high-fat food. *Ox2r*-deficient mice exhibited less energy expenditure than wild-type mice when fed a high-fat diet. Neither *Ox1r*-deficient nor *Ox2r*-deficient mice showed body weight gain similar to *orexin*-deficient mice. Although the presence of a running wheel suppressed diet-induced obesity in wild-type mice, the effect was weaker in orexin neuron-ablated mice. Finally, we did not detect abnormalities in brown adipose tissues of *orexin*-deficient mice. Thus, each orexin receptor signaling has a unique role in energy metabolism, and orexin neurons are involved in the interactive effect of diet and exercise on body weight gain.

## Highlights

• Food intakes of *Ox1r*-deficient mice are similar between chow and high-fat food
• *Ox2r*-deficient mice exhibit less energy expenditure when fed a high-fat diet
• Orexin neurons are involved in the interactive effect of diet and exercise
• *Orexin*-deficient mice have normal brown adipose tissue

## Graphical Abstract

**Figure:**
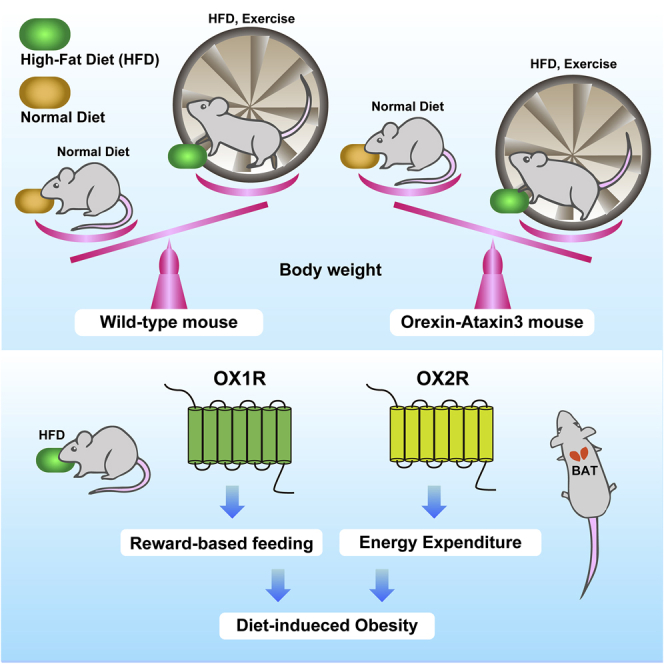

## Introduction

A greater availability of energy-dense foods and reduced physical activity attribute to the current obesity pandemic in the modern society (Article et al., 2017). The C57BL/6 mouse has been used to study diet-induced obesity and to identify the regulatory effect of the gene-environment interaction on body weight (Collins et al., 2004, Funato et al., 2009). Easy access to a high-fat diet causes the mice to become overweight, which has provided insights into the central role of the hypothalamus in energy metabolism. The hypothalamus is composed of a variety of neural groups that are involved in mechanisms regulating feeding, energy expenditure, body temperature, and sleep/wakefulness.

Orexins (also known as hypocretins) are neuropeptides that are expressed in the lateral hypothalamus and were originally identified as factors that enhance feeding behavior (Sakurai et al., 1998). Subsequent research showed a wake-promoting effect of orexins (Chemelli et al., 1999, Sakurai, 2007) and orexin deficiency underlies the pathophysiology of narcolepsy (Nishino, 2007). Counterintuitive to the acute effect of orexin on promoting feeding, orexin deficiency or postnatal ablation of orexin neurons causes obesity in mice (Hara et al., 2005, Hara et al., 2001), suggesting that orexin functions as a negative regulator of energy metabolism. Consistent with these findings, human individuals with narcolepsy have been reported to exhibit a greater body mass index and a higher incidence of metabolic syndrome (Nishino, 2007). In contrast to orexin deficiency, orexin overexpression renders mice resistant to high-fat diet-induced obesity. Among the two receptors for orexin, type 1 (OX1R) and type 2 (OX2R), enhanced OX2R signaling is sufficient to induce resistance to diet-induced obesity in mice (Funato et al., 2009). Although acute or subacute effects of orexin receptor signaling on food intake and energy expenditure has been reported (Funato, 2015, Haynes et al., 2000, Rodgers et al., 2001), very little is known about the roles of the two orexin receptors, particularly their long-term effects on metabolism, partly because the metabolic phenotype of OX1R or OX2R loss-of-function mutant mice has not been reported.

Dual orexin receptor antagonists have been approved as a treatment for insomnia, and researchers are continuing to develop selective antagonists and agonists for each orexin receptor (Coleman et al., 2017, Janto et al., 2018, Nagahara et al., 2015). The development of several other drugs targeting orexin receptors is also underway. Thus, the metabolic characterization of mice deficient in OX1R or OX2R will provide indispensable information about the expected side effects of orexin receptor-targeting drugs and potential therapeutic targets.

Because both the ubiquitous access to a high-fat diet and reduced need for occupational physical activity are two major causes of the increased incidence of human obesity, we examined the effects of the consumption of a high-fat diet and the presence of a running wheel in the home cage on body weight gain in C57BL/6 mice. Since orexin neurons have widespread connections to neural groups regulating energy metabolism and locomotion (Sakurai, 2007), we hypothesize that orexin neurons are involved in the body weight homeostasis through the integrative regulation of food intake and exercise.

In addition to the role of orexin in the central nervous system, orexin is required for the development of brown adipose tissue (BAT) (Sellayah et al., 2011, Sellayah and Sikder, 2014), which may affect metabolic phenotypes. However, we have never noticed gross abnormalities in the BAT of *orexin*-deficient mice, which prompted us to examine the BAT from *orexin*-deficient mice in detail.

Here, we report the different roles of OX1R and OX2R in energy metabolism and glucose metabolism through the metabolic characterization of orexin receptor-deficient mice. We also examined the role of orexin neurons in the interactive effect of diet and exercise on body weight gain. Consistent with our previous observation, we did not detect abnormalities in the morphology of or gene expression in the BAT of *orexin*-deficient mice.

## Results

### Body Weight Gain of Orexin-Deficient and Orexin Receptor-Deficient Mice

**Figure 1:**
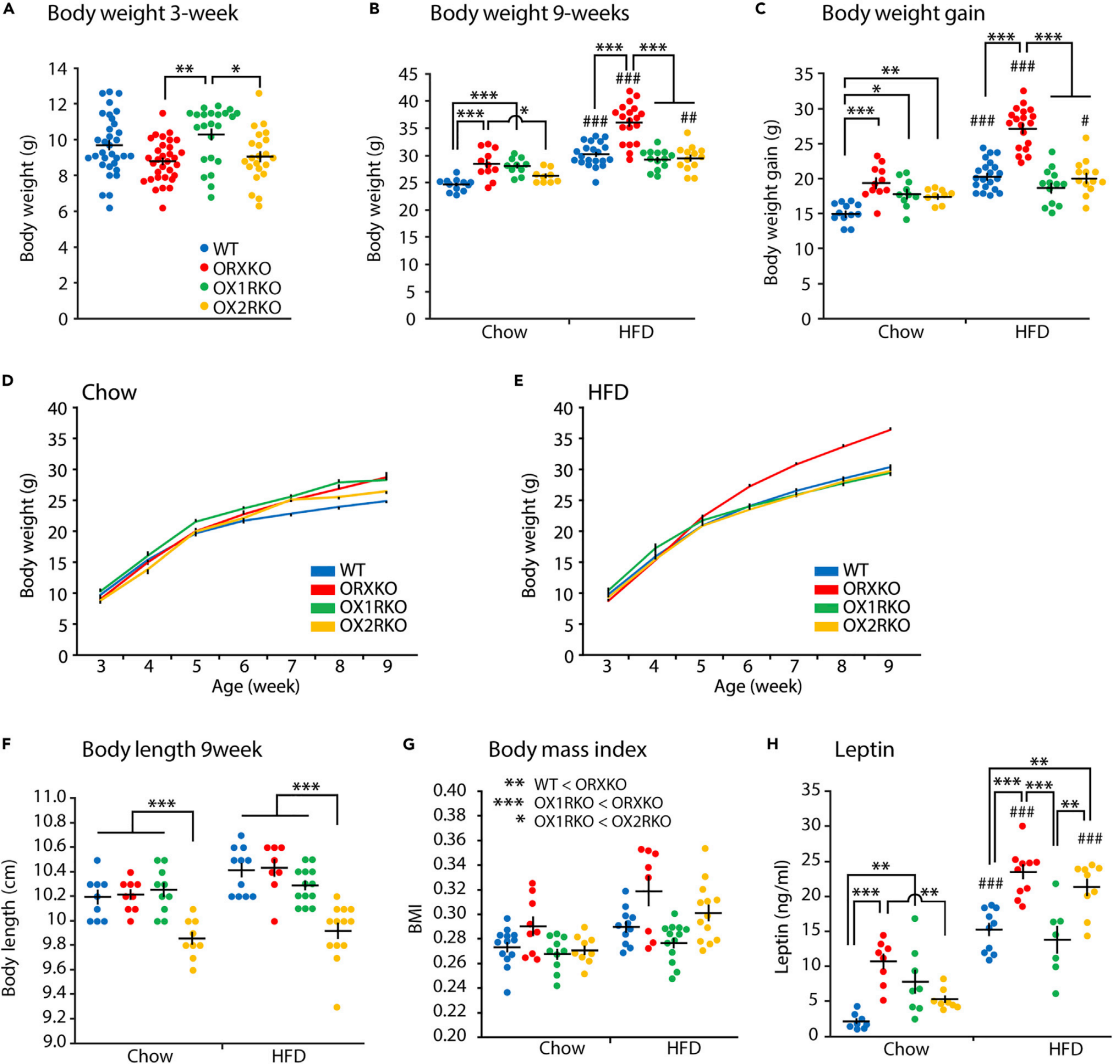
Body Weight Gain of Orexin Receptor-Deficient Mice Fed Chow or High-Fat Diet (HFD) (A) Body weights of wild-type (n = 33), *orexin* KO (n = 32), *Ox1r* KO (n = 23), and *Ox2r* KO mice (n = 22) at the age of 3 weeks. One-way ANOVA followed by Tukey's test. (B–E) Body weight measured at the age of 9 weeks (B) and weight gain from 3 weeks of age (C). Weight gain was measured weekly for chow (D) and HFD (E) mice groups. Wild-type (chow n = 12, HFD n = 21), *orexin* KO (chow n = 13, HFD n = 19), *Ox1r* KO (chow n = 10, HFD n = 13) and *Ox2r* KO mice (chow n = 9, HFD n = 13). (F) At the age of 9 weeks, showing the body lengths of wild-type (chow n = 9, HFD n = 11), *orexin* KO (chow n = 9, HFD n = 8), *Ox1r* KO (chow n = 10, HFD n = 13), and *Ox2r* KO mice (chow n = 9, HFD n = 13). (G) The BMI of wild-type at the age of 9 weeks. Wild-type (chow n = 13, HFD n = 11), *orexin* KO (chow n = 9, HFD n = 8), *Ox1r* KO (chow n = 10, HFD n = 13), and *Ox2r* KO mice (chow n = 9, HFD n = 13). (H) The serum leptin levels in wild-type (chow n = 8, HFD n = 10), *orexin* KO (chow n = 8, HFD n = 10), *Ox1r* KO (chow n = 8, HFD n = 7), and *Ox2r* KO mice (chow n = 8, HFD n = 9). Two-way ANOVA followed by Tukey's test. *p < 0.05, **p < 0.01, ***p < 0.001 among genotypes. #p < 0.05, ##p < 0.01, ###p < 0.001 between diet groups. All data are presented as mean ± SEM. See also Figure S1.

To examine the differential roles of orexin receptors, we used *Ox1r-*deficient mice (Figure S1), *Ox2r*-deficient mice (Willie et al., 2003), and *orexin-*deficient mice (Chemelli et al., 1999). First, we examined the effect of an orexin receptor deficiency on the body weight gain of mice when fed a chow or a high-fat diet. At the age of 3 weeks, when mice were weaned, the body weight of *orexin*-deficient mice tended to be lower than that of wild-type mice, but the difference was not statistically significant (one-way ANOVA, p = 0.02; Tukey's test, p = 0.075, Figure 1A). The body weight of 3-week-old *Ox1r*-deficient mice was greater than that of *orexin*-deficient mice and *Ox2r*-deficient mice (Tukey's test, p = 0.002 for *Ox1r*-deficient mice, p = 0.031 for *Ox2r*-deficient mice, Figure 1A). After weaning, the 3-week-old mice were randomly assigned to regular chow or high-fat diet to determine how orexin signaling affects body weight gain over 6 weeks, which is consistent with the protocol described by Sellayah et al. (2011). *Orexin*-deficient mice fed the regular chow diet displayed a significantly greater body weight at 9 weeks old and greater weight gain over 6 weeks beginning at the age of 3 weeks than wild-type mice (two-way ANOVA, p < 0.001; Tukey's test, p < 0.001, Figures 1B–1D). Both *Ox1r*-deficient and *Ox2r*-deficient mice showed greater weight gains than wild-type mice (Tukey's test, p = 0.004 for *Ox1r*-deficient mice, p = 0.019 for *Ox2r*-deficient mice, Figure 1C).

The body weight of orexin-deficient mice fed a high-fat diet was greater than mice of the other genotypes (Tukey's test, p < 0.001 for each group, Figures 1B, 1C, and 1E). *Orexin*-deficient mice fed a high-fat diet showed greater weight gain from 3 weeks old to 9 weeks old (Tukey's test, p < 0.001 for each group, Figure 1C), consistent with previous reports (Funato et al., 2009, Hara et al., 2005, Sellayah et al., 2011). However, both 9-week-old *Ox1r*-deficient and *Ox2r*-deficient mice showed body weight and weight gain that were similar to that of wild-type mice (Figures 1B and 1C). Interestingly, high-fat diet feeding increased the body weight and weight gain of wild-type, *orexin*-deficient, and *Ox2r*-deficient mice at 9 weeks old compared with animals fed regular chow; the body weight and weight gain of *Ox1r*-deficient mice were similar, regardless of diet, suggesting that *Ox1r*-deficient mice were resistant to diet-induced obesity (Figures 1B and 1C).

At 9 weeks old, the body lengths of *Ox2r*-deficient mice were shorter than mice of other genotypes fed either regular chow or high-fat diet (two-way ANOVA, p < 0.001, Tukey's test, p < 0.001 for between *Ox2r*-deficient and other groups, Figure 1F), which may be associated with reduced bone mass in *Ox2r*-deficient mice (Wei et al., 2014). *Orexin*-deficient mice presented a higher body mass index (BMI) than wild-type and *Ox1r*-deficient mice (p = 0.03 for wild-type; p < 0.001 for *Ox1r*-deficient, Figure 1G). *Ox2r*-deficient mice displayed a higher BMI than *Ox1r*-deficient mice (p < 0.049). Lower serum leptin levels were observed in wild-type mice fed a chow diet than *orexin*-deficient and *Ox1r*-deficient mice (two-way ANOVA, p < 0.001; Tukey's test, p < 0.001 for *orexin*-deficient mice, p = 0.004 for *Ox1r*-deficient mice, Figure 1H), consistent with their body weights (Figure 1B). Lower serum leptin levels were observed in wild-type mice fed a high-fat diet that in *orexin*-deficient and *Ox2r*-deficient mice (p < 0.001 for *orexin*-deficient mice, p = 0.008 for *Ox2r*-deficient mice, Figure 1H), similar to their body weights and BMIs (Figures 1B and 1D). *Ox1r*-deficient mice fed a high-fat diet did not show any significant increase in BMI and leptin levels compared with mice of the same genotype fed a chow diet (Figures 1G and 1H).

### Food Intake of Orexin-Deficient and Orexin Receptor-Deficient Mice

**Figure 2:**
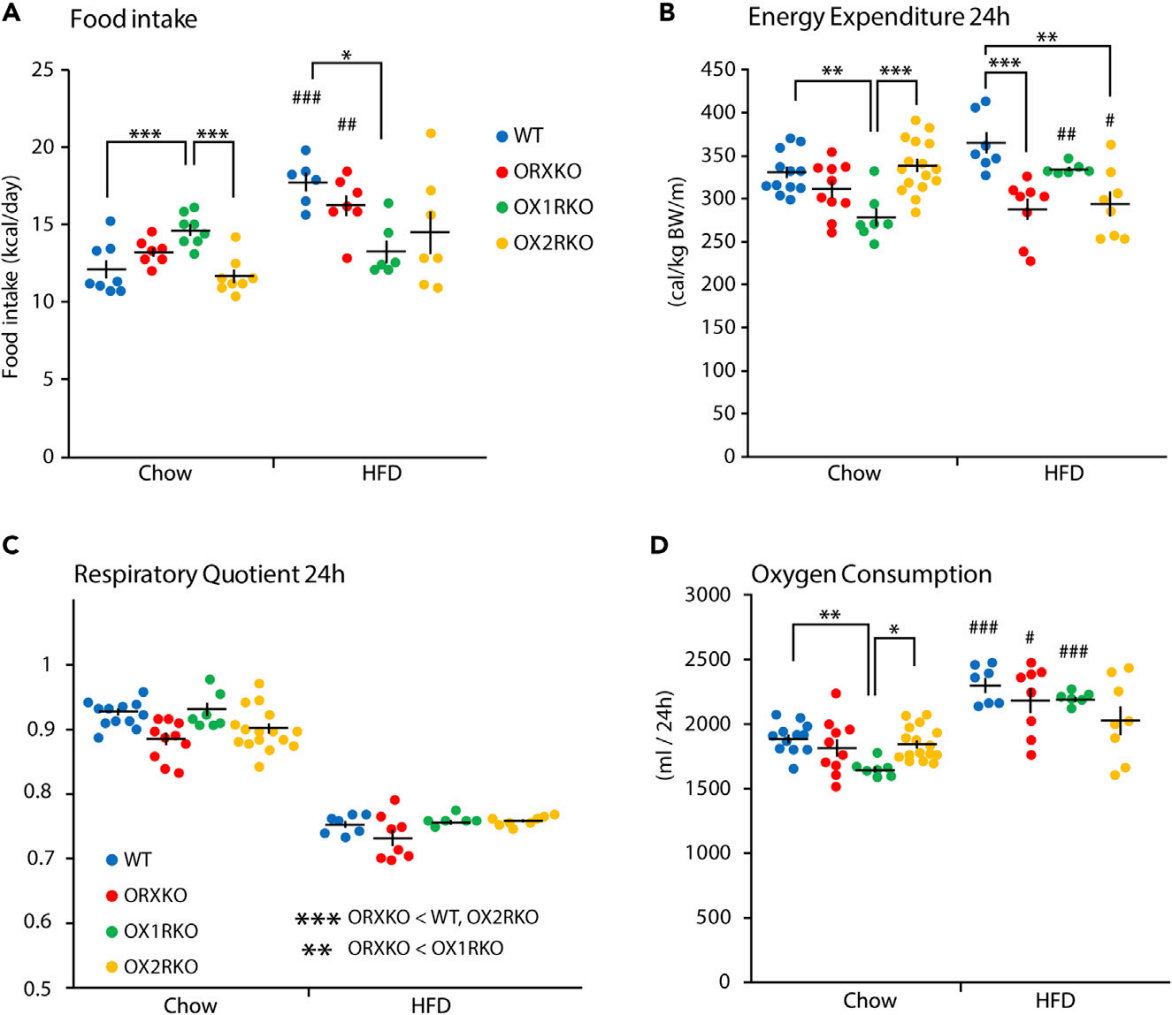
Energy Metabolism of Orexin Receptor-Deficient Mice Fed Chow or High-Fat Diet (HFD) (A) Daily food intake during 11–13 weeks of age in wild-type (chow n = 8, HFD n = 6), orexin KO (chow n = 7, HFD n = 7), *Ox1r* KO (chow n = 8, HFD n = 6), and *Ox2r* KO mice (chow n = 8, HFD n = 7) at the ages of 11–13 weeks. (B–D) The energy expenditure (B), respiratory quotient (C), and oxygen consumption (D) at the ages of 11–13 weeks. The mice were fed a chow or HFD from the age of 3 weeks. Wild-type (chow n = 12, HFD n = 7), *orexin* KO (chow n = 10, HFD n = 8), *Ox1r* KO (chow n = 7, HFD n = 6), and *Ox2r* KO mice (chow n = 16, HFD n = 8). Two-way ANOVA followed by Tukey's test for each diet group. *p < 0.05, **p < 0.01, ***p < 0.001. Compared with the chow diet, #p < 0.05, ##p < 0.01, ###p < 0.001. Data are presented as mean ± SEM.

We examined the daily intake of chow and high-fat diet by mice of each genotype for 4 days at the ages of 11–13 weeks old. The daily chow intake of *orexin*-deficient mice was similar to that of wild-type mice (p = 0.317). *Ox1r*-deficient mice consumed more chow than wild-type mice (p = 0.001) and *Ox2r*-deficient mice (p < 0.001, Figure 2A). In contrast, the *Ox1r*-deficient mice consumed a significantly smaller amount of the high-fat diet than wild-type mice (p = 0.018, Figure 2A). When mice were fed a high-fat diet, wild-type and *orexin*-deficient mice consumed a larger amount of calories from food compared with mice fed a chow diet (Student's t test with Bonferroni correction, p < 0.001 for wild-type, p = 0.002 for *orexin*-deficient mice). When the food intake was compared between mice of the same genotype fed a chow diet and high-fat diet, wild-type and *orexin*-deficient mice consumed larger amounts of calories from the high-fat diet than the chow diet (two-tailed t test, p < 0.001 for wild-type, p = 0.006 for *orexin*-deficient mice). However, a significant difference in the energy intake of *Ox1r*-deficient and *Ox2r*-deficient mice was not observed between chow and the high-fat diet (p = 0.33 for *Ox1r*-deficient mice, p = 0.22 for *Ox2r*-deficient mice, Figure 2A).

### Energy Expenditure of Orexin- or Orexin Receptor-Deficient Mice

We examined the energy expenditure of mice of each genotype fed chow or a high-fat diet. The energy expenditure of *orexin*-deficient mice fed a chow diet was similar to that of wild-type mice. *Ox1r*-deficient mice showed lower energy expenditure than wild-type and *Ox2r*-deficient mice (p = 0.002 for wild-type mice, p < 0.001 for *Ox2r*-deficient mice, Figure 2B). On a high-fat diet, wild-type mice expended more energy than *orexin*-deficient and *Ox2r*-deficient mice (p < 0.001 for wild-type mice, p = 0.002 for *Ox2r*-deficient mice, Figure 2B). Although *Ox1r*-deficient mice showed increased energy expenditure on a high-fat diet compared with the chow diet (two-tailed t test with Bonferroni correction, p = 0.002), *Ox2r*-deficient mice showed decreased energy expenditure (p = 0.025). Both wild-type and *orexin*-deficient mice did not show any significant change in energy expenditure between regular chow and the high-fat diet (p = 0.07 for wild-type; p = 0.62 for *orexin*-deficient).

The respiratory quotient of *orexin*-deficient mice was lower than that of wild-type (two-way ANOVA, p < 0.001; Tukey's test, p < 0.001), *Ox1r*-deficient (p = 0.005), and *Ox2r*-deficient mice (p < 0.001). Mice of all genotypes fed a high-fat diet showed a significant decrease in the respiratory quotient compared with mice fed regular chow (p < 0.001 for all groups, Figure 2C). The oxygen consumption of *orexin*-deficient mice fed a chow diet was similar to that of wild-type mice. *Ox1r*-deficient mice showed lower oxygen consumption than the other groups (p = 0.006 for wild-type mice, p = 0.022 for *Ox2r*-deficient mice, Figure 2D). A significant difference in oxygen consumption was not observed among the different mouse strains after feeding on a high-fat diet (one-way ANOVA, p = 0.17). Wild-type, *orexin*-deficient, and *Ox1r*-deficient mice showed increased oxygen consumption on a high-fat diet than on a chow diet (two-tailed t test with Bonferroni correction, p < 0.001 for wild-type, p = 0.02 for *orexin*-deficient and p < 0.001 for *Ox1r*-deficient mice, Figure 2D). In contrast, *Ox2r*-deficient mice did not show any change in oxygen consumption between chow and a high-fat diet (p = 0.21 for *Ox2r*-deficient mice).

### Glucose Metabolism in Orexin- or Orexin Receptor-Deficient Mice

**Figure 3:**
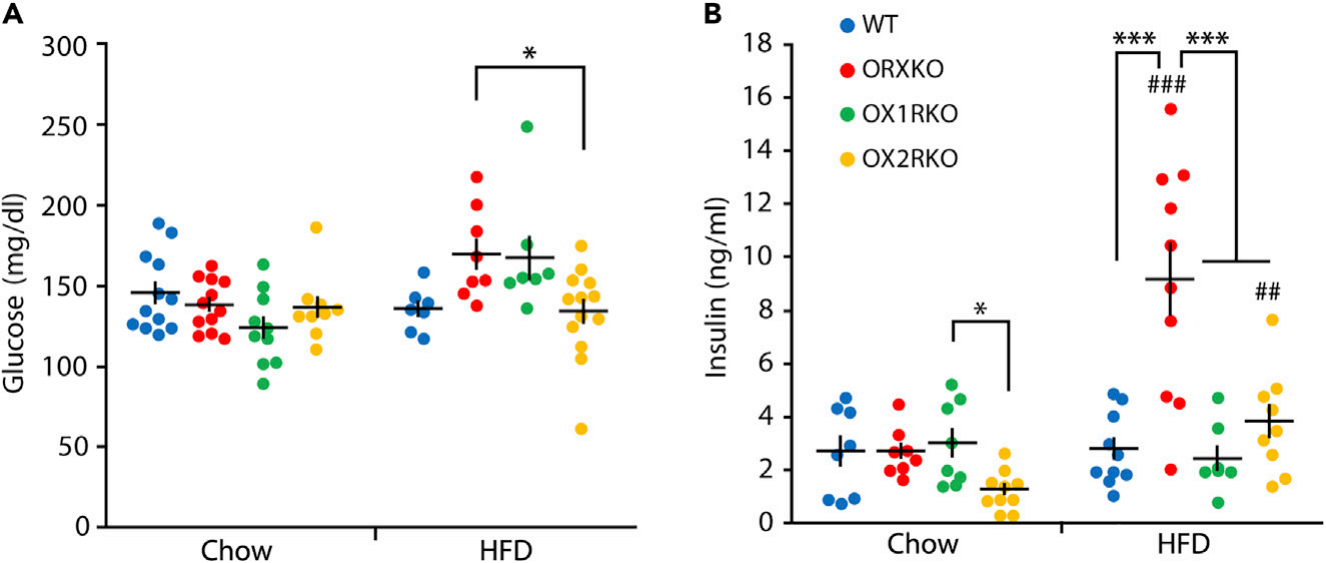
Glucose Metabolism of Orexin Receptor-Deficient Mice Fed Chow or High-Fat Diet (HFD) (A) The blood glucose levels of wild-type (chow n = 12, HFD n = 7), *orexin* KO (chow n = 12, HFD n = 8), *Ox1r* KO (chow n = 10, HFD n = 7), and *Ox2r* KO mice (chow n = 9, HFD n = 13) at the age of 9 weeks. (B) The serum insulin levels in wild-type (chow n = 8, HFD n = 10), *orexin* KO (chow n = 8, HFD n = 10), *Ox1r* KO (chow n = 8, HFD n = 7), and *Ox2r* KO mice (chow n = 10, HFD n = 9). Two-way ANOVA followed by Tukey's test for each diet group. *p < 0.05, ***p < 0.001. Compared with the chow diet, ##p < 0.01, ###p < 0.001. Data are presented as mean ± SEM.

Next, we examined glucose metabolism in mice deficient in orexin and its receptors. Nine-week-old *orexin*-deficient and orexin receptor-deficient mice fed a chow diet displayed a similar glucose level to wild-type mice (Figure 3A). A lower serum insulin level was observed in *Ox2r*-deficient mice than in *Ox1r*-deficient mice (One-way ANOVA, p = 0.02; Tukey's test, p = 0.03, Figure 3B). After consumption of a high-fat diet, a higher glucose level was observed in *orexin*-deficient mice than in *Ox2r*-deficient mice (p = 0.041, Figure 3A). A higher insulin level was observed in *orexin*-deficient mice than in the other animal groups (p < 0.001 for all groups, Figure 3B). Although higher insulin levels were detected in *orexin*-deficient and *Ox2r*-deficient mice fed a high-fat diet than in animals fed a chow diet (two-tailed Student t test with Bonferroni correction, p < 0.001 for *orexin*-deficient, p = 0.004 for *Ox2r*-deficient mice), wild-type and *Ox1r*-deficient mice did not show significant differences in insulin levels between the regular chow and high-fat diet (Figure 3B).

### Hypothalamic Gene Expression in Orexin- or Orexin Receptor-Deficient Mice

**Figure 4:**
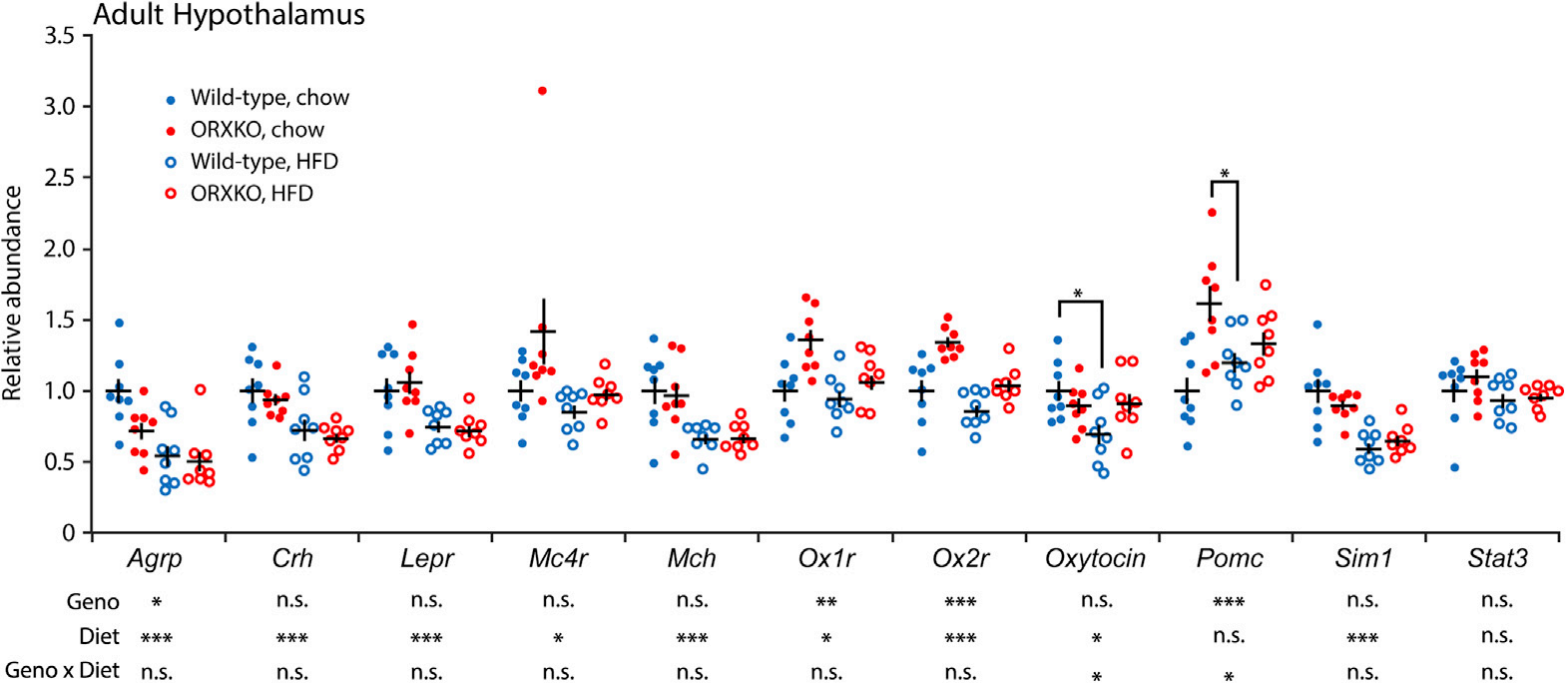
Gene Expressions in Hypothalamus from Adult *Orexin*-Deficient Mice The expression is presented relative to the mean of wild-type mice that fed the chow diet. Two-way ANOVA followed by Tukey's test for each diet group. Ten mice per group. *p < 0.05, **p < 0.01, ***p < 0.001. Data are presented as mean ± SEM.

We examined how orexin deficiency and high-fat diet feeding alter the expression of genes related to food intake and energy metabolism in the adult hypothalamus. A two-way ANOVA identified suppressive effects of high-fat diet feeding on *Agrp*, *Crh*, *Lepr*, *Mc4r*, *Mch*, *Ox1r*, *Ox2r*, and *Sim1* expression (Figure 4). *Orexin*-deficient mice showed reduced expression of *Agrp*, *Ox1r*, and *Ox2r*. Wild-type mice fed a high-fat diet showed decreased expression of *oxytocin* compared with animals fed a chow diet. Chow diet-fed *orexin*-deficient mice expressed *Pomc* at higher levels than wild-type mice.

### Reduced Anti-obesity Effect of Exercise in Orexin Neuron-Ablated Mice

**Figure 5:**
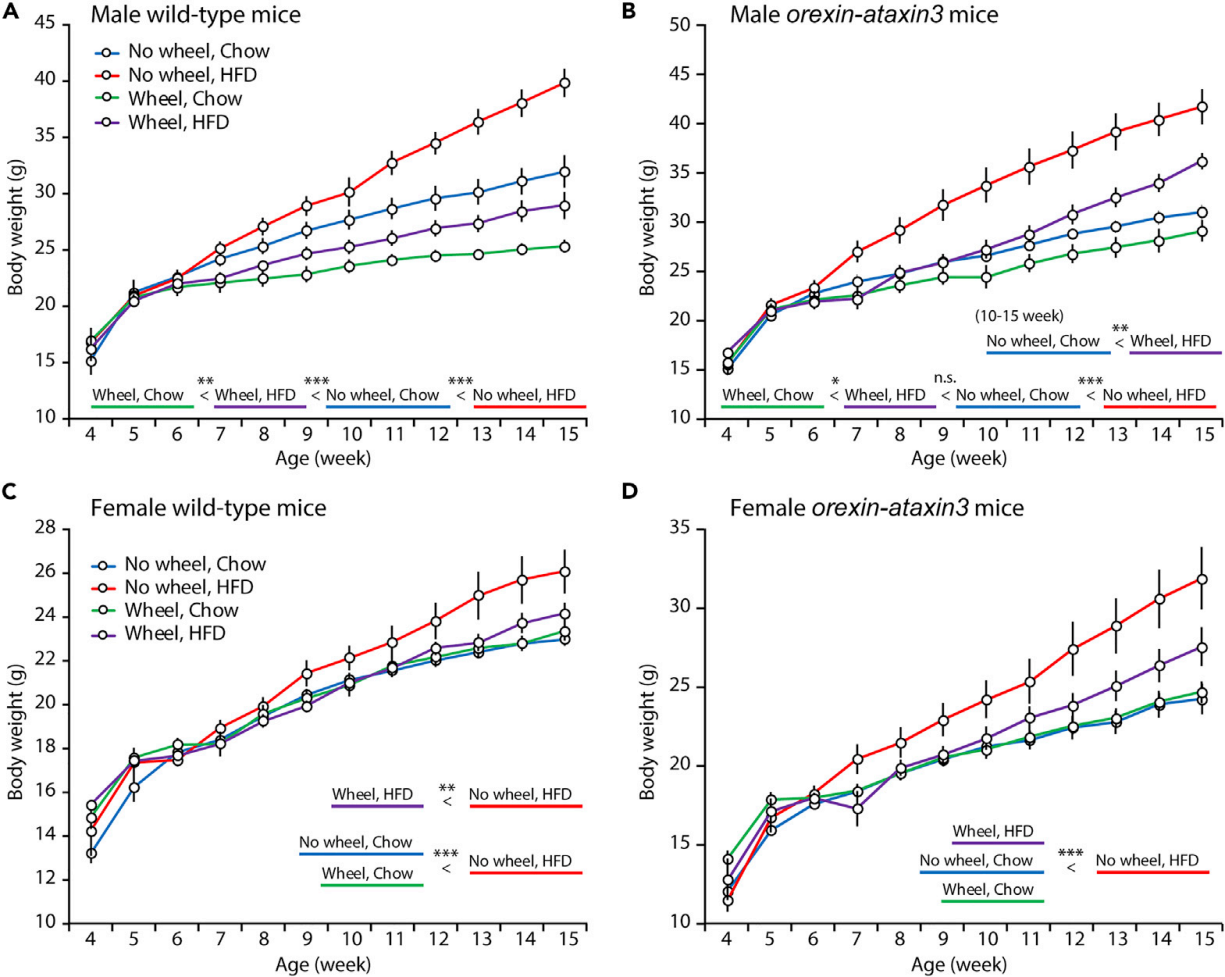
Effects of Exercise and a High-Fat Diet on the Body Weight Growth of Orexin Neuron-Ablated Mice (A) Male wild-type mice. Chow without a wheel (n = 12), high-fat diet (HFD) without a wheel (n = 11), chow with a wheel (n = 7), HFD with a wheel (n = 12). (B) Male *orexin-ataxin3* mice. Chow without a wheel (n = 10), HFD without a wheel (n = 9), chow with a wheel (n = 8), HFD with a wheel (n = 7). (C) Female wild-type mice. Chow without a wheel (n = 20), HFD without a wheel (n = 10), chow with a wheel (n = 9), HFD with a wheel (n = 10). (D) Female *orexin-ataxin3* mice. Chow without a wheel (n = 7), HFD without a wheel (n = 8), chow with a wheel (n = 8), HFD with a wheel (n = 6). For (A)–(D) body weight was measured weekly from the ages of 4 to 15 weeks. Mice were fed normal chow or a high-fat diet and housed in a cage equipped with or without a running wheel. Two-way ANOVA followed by a comparison of the main effect after Bonferroni adjustment. *p < 0.05, **p < 0.01, ***p < 0.001. Data are presented as mean ± SEM.

Considering the rapid increase in the obese population in modern society due to the availability of cheap and high-caloric food and the decreased need for involuntary exercise, we examined the body weight gain of mice, which were group-housed in cages equipped with or without a running wheel and fed a normal chow or a high-fat diet from weaning to the age of 15 weeks. The presence of the running wheel suppressed the body weight gain of wild-type mice fed either regular chow or a high-fat diet (Figure 5A). Importantly, male mice fed a high-fat diet in a cage equipped with a running wheel gained less body weight than mice fed a chow diet, suggesting that the suppressive effect of a running wheel surpasses the obesogenic effect of the high-fat diet on wild-type mice. We used *orexin-ataxin3* mice in which orexin neurons are ablated in the early postnatal period (Hara et al., 2001) to avoid possible developmental perturbation due to the loss of orexin signaling and the progressive effect of the loss of orexinergic neurons. Similar to wild-type mice, *orexin-ataxin3* mice exhibited high-fat diet-induced obesity, and the presence of a running wheel suppressed this effect (Figure 5B). In contrast to wild-type mice, orexin neuron-ablated male mice fed a normal chow in a cage without a running wheel gained less body weight than mice fed a high-fat diet in a cage with a running wheel after the age of 10 weeks, when almost all orexin neurons were ablated (Hara et al., 2001). Based on this result, the suppressive effect of a running wheel is weaker in male orexin neuron-ablated mice. High-fat diet feeding increased body weight gain in female wild-type mice to a lesser extent than in male mice, consistent with the tight homeostatic regulation of body weight in female mice (Funato et al., 2009). A running wheel exerted a suppressive effect on weight gain in female mice fed a high-fat diet but not a chow diet (Figure 5C). Similarly, a similar suppressive effect of the running wheel was only observed in female orexin neuron-ablated mice fed a high-fat diet (Figure 5D).

### BAT of Orexin- and Orexin Receptor-Deficient Mice

**Figure 6:**
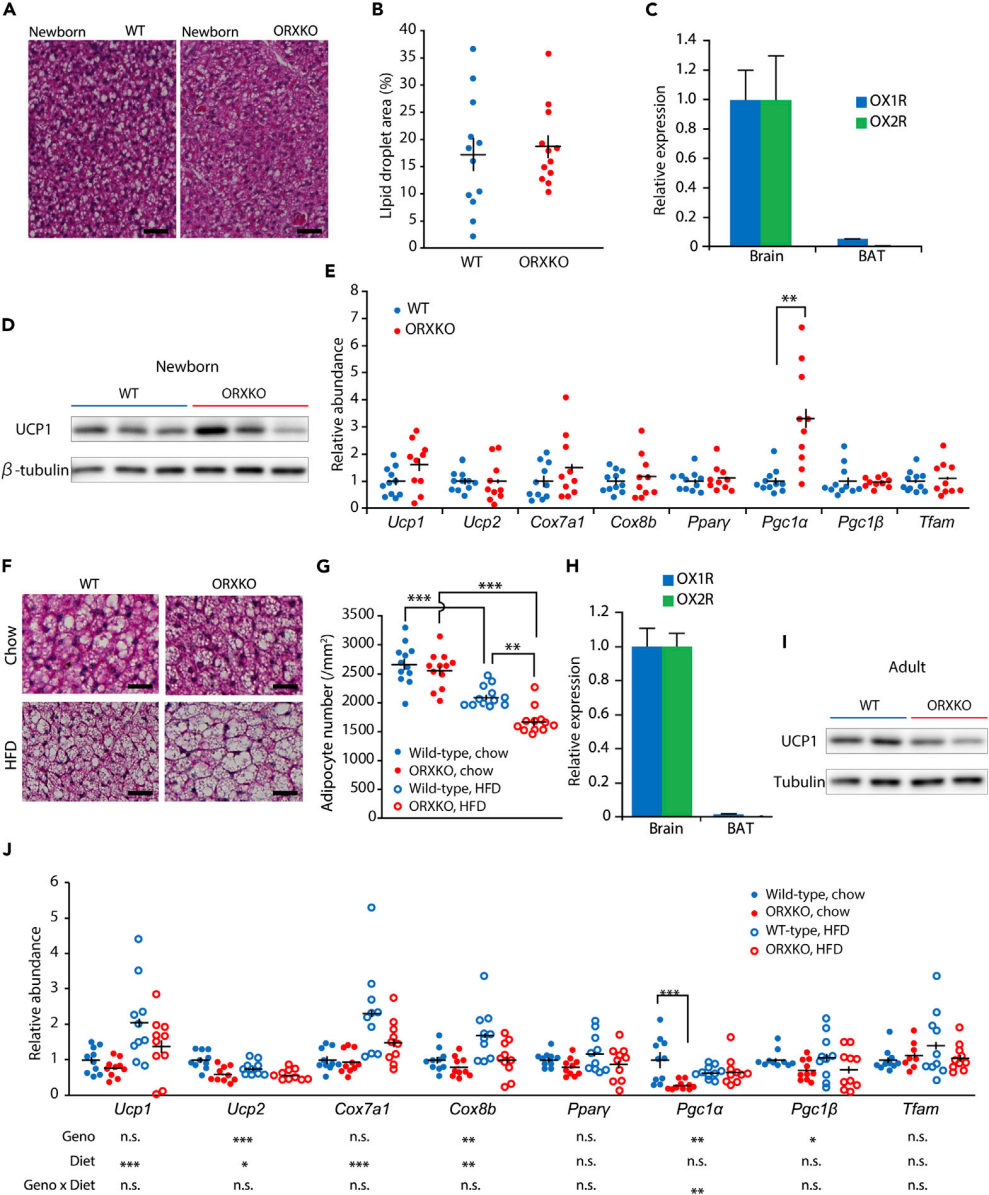
Histology and Gene Expression in the BAT of *Orexin*-Deficient Mice (A) Representative images of BAT stained with hematoxylin and eosin from newborn wild-type (WT) and *orexin*-deficient (ORXKO) mice. Scale bars, 40 μm. (B) Lipid droplet areas of the BAT in newborn WT and ORXKO mice (12 mice per group). (C) Expression of orexin receptor genes in BAT from newborn wild-type mice (n = 7). The expressed is presented relative to the mean of orexin receptor expression in the brain. (D) Western blot images of UCP1 and β-tubulin in BAT of newborn WT and ORXKO mice. (E) Expression of genes related to the function and differentiation of BAT in newborn WT mice (n = 11) and ORXKO (n = 10). The expression is presented relative to the mean of newborn WT mice. **p < 0.01 obtained using t test with Bonferroni correction. (F) Representative images of BAT stained with hematoxylin and eosin from adult mice. Scale bars, 40 μm. (G) Adipocyte numbers of the BAT in adult male WT and ORXKO mice fed normal chow or on HFD (12 mice for each group). (H) Expression of orexin receptor genes in BAT from adult wild-type mice fed normal chow (n = 6, 4–6 months old). The expression is presented relative to the mean orexin receptor expression in the brain. *Ox2r* was not detected in BAT. (I) Representative western blot images of UCP1 and β-tubulin in BAT of chow-fed 14-week-old WT and ORXKO mice. (J) Expression of genes related to the function and differentiation of BAT in adult male WT and ORXKO mice, which were fed normal chow or HFD (n = 10 for each group). The expression is presented relative to the mean of WT mice fed normal chow. *p < 0.05, **p < 0.01, ***p < 0.001. All data are presented as mean ± SEM. See also Figure S2.

Although many studies have focused on the central actions of orexin in energy metabolism (Funato, 2015, Sakurai and Mieda, 2011), Sellayah et al. reported the maldevelopment of the BAT in *orexin*-deficient mice as early as the neonatal period, which may lead mice to become overweight (Sellayah et al., 2011). However, the BAT of newborn *orexin*-deficient mice was indistinguishable from newborn wild-type mice, regardless of whether their mothers were heterozygous or homozygous *orexin*-deficient mice (Figure 6A). In contrast to the findings of the previous report (Sellayah et al., 2011), no difference in lipid droplet areas was observed between wild-type and *orexin*-deficient mice (Figures 6B and S2). Very low or undetectable levels of orexin receptors were expressed in the BAT of newborn wild-type mice (Figure 6C). No difference in UCP1 protein level in the BAT was observed between newborn wild-type and *orexin*-deficient mice (Figure 6D). Among the genes associated with the function and development of the BAT, such as *Ucp1*, *Ucp2*, *Cox7a1*, *Cox8b*, *Pparγ*, *Pgc1α*, *Pgc1β*, and *Tfam*, only *PGC1α* was upregulated in the BAT of newborn *orexin*-deficient mice (two-tailed Student's t test, p < 0.001, Figure 6E). This observation is opposite to the previous results (Sellayah et al., 2011). Thus, newborn *orexin*-deficient mice showed normal histological and molecular characteristics of the BAT.

When adult mice were fed a chow diet, the BAT of *orexin*-deficient mice was similar to that of wild-type mice (Figure 6F). The BAT of *orexin*-deficient mice fed a high-fat diet displayed a decrease in the cell density compared with wild-type mice owing to a larger cell size that was enriched in lipid droplets (Figure 6G), which may have resulted from the obesity of *orexin*-deficient mice. Similar to newborn pups, adult BAT expressed a very low level of OX1R and almost undetectable level of OX2R (Figure 6H). UCP1 protein level in the BAT of adult *orexin*-deficient mice was lower than that of wild-type mice (Figure 6I). Two-way ANOVA detected a significant effect of high-fat diet feeding on increased *Ucp1*, *Ucp2*, *Cox7a1*, and *Cox8b* expression (Figure 6J). *Orexin*-deficient mice showed decreased *Ucp2*, *Cox8b*, and *Pgc1β* expression. *Orexin*-deficient mice fed a chow diet showed reduced *Pgc1α* expression.

## Discussion

Based on the findings from the current study, each orexin receptor has characteristic roles in energy metabolism and the diet-induced obesity of *orexin*-deficient mice is unlikely to be attributed to the lack of signaling from a single orexin receptor.

*Orexin*-deficient mice showed higher body weight and leptin levels at 9 weeks old and a greater weight gain from 3 weeks old to 9 weeks old than wild-type mice, regardless of diet. The current results confirmed previous reports that *orexin*-deficient mice are susceptible to diet-induced obesity (Hara et al., 2005, Sellayah et al., 2011). However, neither *Ox1r*-deficiency nor *Ox2r*-deficiency mimicked the strong diet-induced obesity observed in *orexin*-deficient mice, suggesting that deficiency in a single receptor signaling is not sufficient to render mice more susceptible to diet-induced obesity. Similar to the metabolic characteristics, neither *Ox1r*-deficient or *Ox2r*-deficient mice showed sleep abnormalities, such as frequent cataplexy-like behavior, observed in *orexin*-deficient mice (Chemelli et al., 1999, Hasegawa et al., 2014, Mahoney et al., 2019, Willie et al., 2003). Although *orexin*-deficient mice showed high-fat diet intake similar to wild-type mice, *orexin*-deficient mice showed lower energy expenditure than wild-type mice, which may be associated with lower expression of *Ucp2*, *Cox8b*, and *Pgc1β* in the BAT. *Orexin*-deficient mice also showed lower UCP1 protein in the BAT despite no change in *Ucp1* mRNA, suggesting fast UCP1 degradation in *orexin*-deficient mice. Thus, insufficient energy usage may underlie the higher susceptibility of *orexin*-deficient mice to diet-induced obesity. Although we did not examine in this study, another possible target of the orexin system is creatine-driven futile cycling in beige fat, which is largely independent of UCP1 (Kazak et al., 2015).

Our data suggest an inverse relationship between the body weight gain and energy expenditure in wild-type and *orexin*-deficient mice. *Orexin*-deficient mice gained 4 g of body weight as shown in Figure 1C and decreased energy expenditure by around 20 cal/kg/BW/m (Figure 2B) in 6 weeks when compared with the wild-type mice. The body weight gain can be translated into fat mass growth of 4 g (36 kcal), equivalent to the cumulative sum of the energy expenditure reduction over 6 weeks. However, the change in energy expenditure value was not statistically significant between the wild-type and *orexin*-deficient mice. This could be due to the larger variability in energy expenditure than the body weight that may lead to the lower statistical power, thus failing to reach statistical significance. In addition, although orexin was originally identified as an orexigenic peptide (Sakurai et al., 1998), the effect is acute and recognized during the light phase. Long-term injection of orexin did not change food intake (Yamanaka et al., 1999), and dual orexin receptor agonist did not alter food intake (Tsuneki et al., 2016). Consistently, we observed similar food intake between wild-type and orexin-deficient mice.

**Figure 7:**
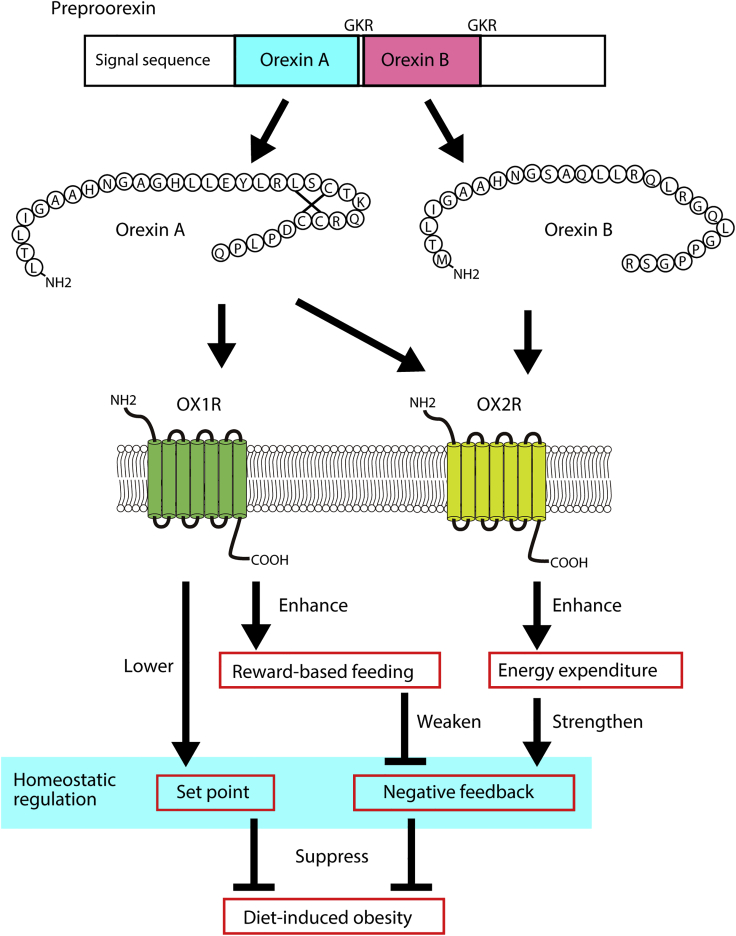
Different Roles of Orexin Receptors on Energy Metabolism Mainly Based on the Current Study

*Ox1r*-deficient mice showed greater body weight and higher leptin levels at 9 weeks old and a greater weight gain from 3 weeks old to 9 weeks old than wild-type mice when fed a chow diet, which mimics the metabolic phenotype of *orexin*-deficient mice fed a chow diet. When *Ox1r*-deficient mice were fed a high-fat diet, however, the body weight and leptin levels at 9 weeks old and weight gain from 3 weeks old to 9 weeks old were similar to that of wild-type mice and significantly lower than that of *orexin*-deficient mice. Importantly, high-fat diet feeding did not increase the body weight and leptin levels of *Ox1r*-deficient mice. Thus, *Ox1r*-deficient mice were characterized by resistance to diet-induced obesity and a higher body weight set point (Figure 7). The tight regulation of energy balance in *Ox1r*-deficient mice underlies the stable caloric intake between chow and high-fat diet feeding, and the increased energy expenditure when fed a high-fat diet compared with regular chow. In addition, the loss of OX1R signaling may disturb reward-related high-fat diet intake that is observed in wild-type C57BL/6 mice (Johnson and Kenny, 2010). The lack of increase in the intake of the high-fat diet by *Ox1r*-deficient mice suggests a disturbance in the reward behavior (Sakurai, 2014), consistent with the effect of OX1R on dopaminergic neurons (Moorman and Aston-Jones, 2010, Prince et al., 2015), and the suppression of motivation for high-fat diet feeding by an OX1R antagonist (Alcaraz-Iborra et al., 2014, Borgland et al., 2009, Nair et al., 2008, Sharf et al., 2010, Steiner et al., 2013, Valdivia et al., 2014), but not by an OX2R antagonist (Piccoli et al., 2012).

*Ox2r*-deficient mice displayed greater weight gain from 3 weeks old to 9 weeks old than wild-type mice when fed a chow diet, similar to *orexin*-deficient mice and *Ox1r*-deficient mice. When fed a high-fat diet, *Ox2r*-deficient mice showed a higher leptin level than wild-type mice and a similar body weight to wild-type mice, which may partially be explained by the shorter body length of *Ox2r*-deficient mice. In contrast to *Ox1r*-deficient mice, high-fat diet feeding increased the body weight and leptin levels of *Ox2r*-deficient mice. *Ox2r*-deficient mice were characterized by reduced energy expenditure when fed a high-fat diet compared with regular chow (Figure 6). Thus, OX2R signaling is required for the proper response to a high-fat diet by increasing energy expenditure and is consistent with the role of OX2R signaling as a negative regulator of the energy balance, based on an orexin overexpression study (Funato et al., 2009).

High-fat diet feeding increased serum insulin levels in *orexin*-deficient mice and *Ox2r*-deficient mice. Since an increased serum insulin level is an early indicator of a deterioration of glucose metabolism, the disturbed glucose metabolism in *orexin*-deficient mice and *Ox2r*-deficient mice is largely consistent with the body mass index and leptin levels. Thus, although orexin receptors are expressed in the pancreatic islet (Adeghate et al., 2010, Kirchgessner and Liu, 1999, Nowak et al., 2005), the absence of the orexin receptors did not exert an apparent effect on glucose metabolism.

The differential effects of OX1R- and OX2R-signaling may be attributed to the biased expression of each orexin receptor in neuronal groups and also to different intracellular signaling. We recently showed that high-fat diet-fed orexin-overexpression mice exhibited increased phosphorylation level of S6 and S6K, indicators of the mTORC1 pathway activity, compared with high-fat-fed wild-type mice (Wang et al., 2014). Considering that orexin-overexpression mice are resistance to diet-induced obesity via OX2R signaling (Funato et al., 2009), the OX2R-mTORC1 pathway may be involved in energy expenditure regulation. Since mTORC1 plays important roles in nutrient sensing, glucose homeostasis, and energy metabolism (Saxton and Sabatini, 2017), mTORC1 may expand the diversity in the orexin receptor downstream effectors, in addition to the well-known downstream cascade involving effectors like PKA and PKC (Wang et al., 2018).

As shown in the current study, the presence of a running wheel in the home cage suppressed high-fat diet-induced weight gain in both male and female mice. The suppressing effect of a running wheel exceeded the obesogenic effect of the consumption of a high-fat diet in male wild-type mice. In contrast, the presence of the running wheel exerted a weaker suppressive effect on male orexin neuron-ablated mice, as the body weight of mice fed a high-fat diet in a cage equipped with a running wheel group was greater than that of mice fed a chow diet in a cage without a running wheel. This finding implies that orexin neurons are involved in the homeostatic body weight regulation through a coordinated modulation of high-fat diet intake and exercise.

Although Sellayah et al. claimed that peripheral orexin functions in the development and maintenance of normal BAT (Sellayah et al., 2011, Sellayah and Sikder, 2014), we failed to reproduce their main findings, such as low levels of intracellular lipid droplet accumulation and decreased expression of genes related to mitochondrial energy production, in the BAT of neonatal and adult mice. Since the authors did not show diet-induced obesity in wild-type C57BL/6 mice (Figure 1A in Sellayah et al., 2011), despite numerous reports (Collins et al., 2004, Funato et al., 2009) including the current study produced the results (Figure 1), we suspect that their study had some technical issues. Thus, the current results do not support the direct effect of orexin on BAT development but, instead, the role of orexin signaling in the regulation of BAT via sympathetic system. Importantly, Martin et al. showed that the ventromedial hypothalamus functions as upstream of orexin neurons, which in turn activates BAT via VGlut2-dependent sympathetic pathway (Martins et al., 2016). The raphe pallidus is the important relay that receives orexin input and promotes thermogenesis of the BAT (Tupone et al., 2011).

Another finding reported in the present study is the short body length of *Ox2r*-deficient mice, which may be associated with the low body mass of *Ox2r*-deficient mice (Wei et al., 2014). Further studies are necessary to explain why *orexin*-deficient mice have normal body length despite the loss of OX2R signaling. One hypothesis is that the suppressive effect of the loss of OX2R signaling on body length growth may depend on OX1R signaling.

In conclusion, OX1R and OX2R signaling are involved in different aspects of energy metabolism and deficiency in both receptors may be required to develop the diet-induced obesity observed in *orexin*-deficient mice. We also examined the role of orexin neurons in the effect of the interaction between diet and exercise on body weight gain. Finally, we did not detect abnormalities in the morphology of or gene expression in the BAT of *orexin*-deficient mice. Thus, we postulate that the central action of orexin underlies the susceptibility of *orexin*-deficient mice to obesity and that the BAT develops normally even in the absence of orexin. This study strongly suggests that orexin receptors are attractive drug targets as treatment for obesity by reducing reward value of energy-dense palatable food and increasing energy expenditure. Notably, orexin neurons functions as an integrative center to shape behaviors.

### Limitations of the Study

In our study, we demonstrate the differential roles of each orexin receptor in food intake and energy expenditure using young adult mice. As aging affects energy and glucose metabolism, the metabolic phenotypes of orexin receptor-deficient mice may vary depending on the age. In previous study, for example, we showed lower insulin level in OX1R-deficient mice in older mice (Funato et al., 2009).

Since OX1R and OX2R are expressed in the brain with some anatomical differences (Mieda and Sakurai, 2011), future work is necessary to identify brain regions or neuronal groups that are responsible for the differential metabolic effect of each orexin receptor. In addition, as orexin receptors are expressed in the peripheral tissues (Xu et al., 2013), the peripheral action of orexin may exert its effect through the maintenance of the intestinal barrier (Tunisi et al., 2019).

## Methods

All methods can be found in the accompanying Transparent Methods supplemental file.

## Supplemental Information

Document S1. Transparent Methods and Figures S1 and S2
