# Supplementary material for: Differential Roles of Each Orexin Receptor Signaling in Obesity
Source: iScience. 2019 Sep 9;20:1–13. doi: 10.1016/j.isci.2019.09.003 (PMC6817686; doi:10.1016/j.isci.2019.09.003)
Supplement: Document S1. Transparent Methods and Figures S1 and S2 [file mmc1.pdf]

ISCI, Volume *20*

Supplemental Information

Differential Roles of Each Orexin

Receptor Signaling in Obesity

Miyo Kakizaki, Yousuke Tsuneoka, Kenkichi Takase, Staci J. Kim, Jinhwan Choi, Aya Ikkyu, Manabu Abe, Kenji Sakimura, Masashi Yanagisawa, and Hiromasa Funato

Supplemental Figures

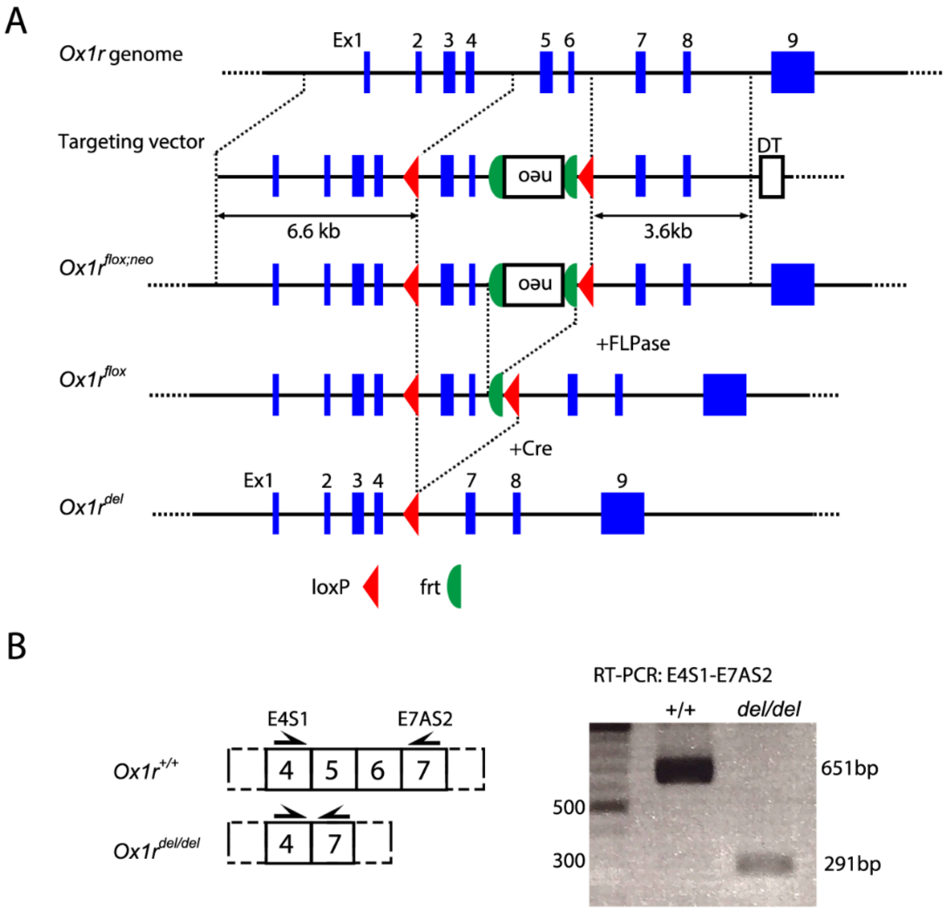

Figure S1

Production of *Ox1r*-floxed and *Ox1r*-deficient mice, Related to Figure 1.

(A) Schematic diagram of the *Ox1r* genome, targeting vector and $Ox1r^{flox;\ neo}$ allele. The PGK-Neo cassette was deleted by $\beta\text{-}actin^{FLPase/+}$ to produce the $Ox1r^{flox}$ allele. The cross with a systemic Cre recombinase driver mouse $Ayu\text{-}1^{Cre/+}$ produced the $Ox1r^{del}$ allele. DT; diphtheria toxin. **(B)** Genotyping for $Ox1r^{del/del}$ mouse. Primer set E4S1/E7As2 captures band at 651 bp for the wild type allele and 291 bp for the $Ox1r^{de/del}$ allele.

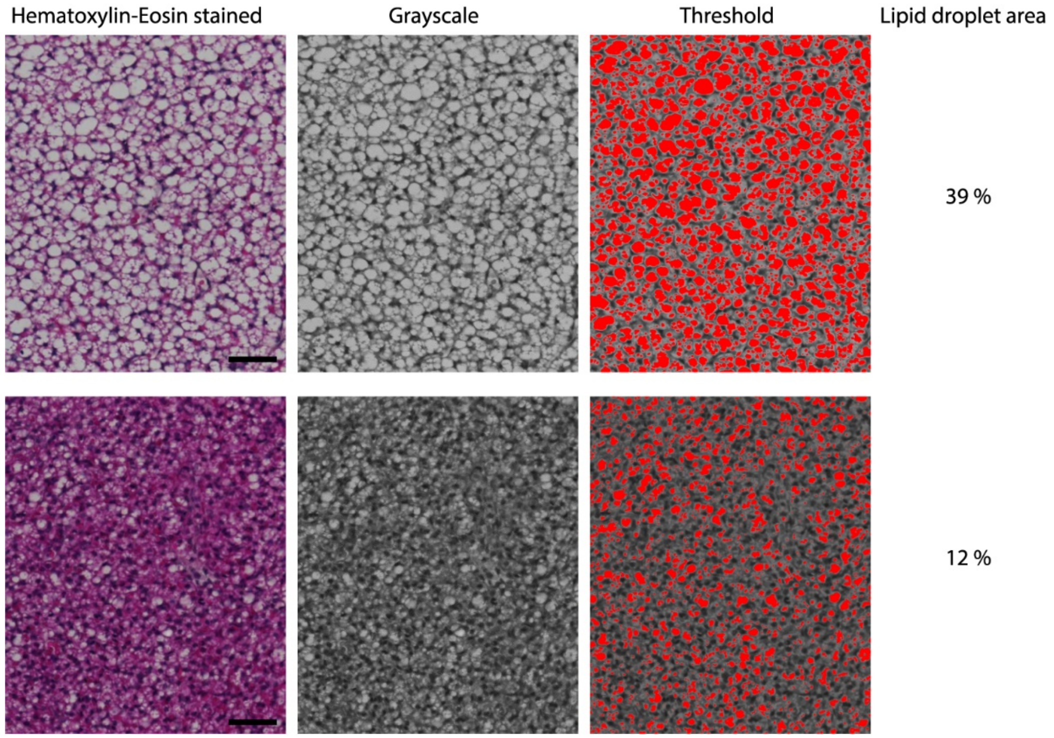

Figure S2. Assessment of lipid droplets in BAT of newborn mice, Related to Figure 6

Images of hematoxylin-eosin-stained sections (left column) of newborn wild-type mice were converted to grayscale (middle column) and further thresholded with a size filter (right column). The upper and lower rows show images of tissues containing lipid droplet areas of 39% and 12%, respectively. Scale bars: 50 μm.

Transparent Methods

Animals

Male C57BL/6J mice (CLEA Japan, Tokyo, Japan) were used in this study. Mice were provided food and water ad libitum, maintained on a 12-hour light/dark cycle and housed under controlled temperature and humidity conditions. All procedures were conducted in accordance with the Guidelines for Animal Experiments of University of Tsukuba and Toho University, and were approved by the Institutional Animal Care and Use Committee of University of Tsukuba (Approved protocol ID # 150194) and Toho University (Approved protocol ID # 18-42-349). We used *orexin*-deficient mice (Chemelli et al., 1999), *Ox2r*-deficient mice (Willie et al., 2003) and *orexin-ataxin* transgenic mice (Hara et al., 2001). *Ox1r*-deficient mice are described below.

Production of *Ox1r*-deficient mice

A genomic fragment containing exons 5 (244 bp) and exon 6 (116 bp) of the *Ox1r* gene was isolated from a C57BL/6 mouse genomic BAC clone from a RP23 mouse genomic BAC library (Advanced GenoTEchs Co) to generate *Ox1r*-deficient mice. Exons 5 and 6 encode the region from the third transmembrane domain through the fifth transmembrane domain. A 1.7-kb fragment of the FRT-PGK-gb2-neo-FRT-loxP cassette (Gene Bridges) was inserted after exon 6. The targeting vector also contains a loxP sequence before exon 5. The targeting vector was linearized and electroporated into the C57BL/6N ES cell line RENKA. Correctly targeted clones were injected into eight-cell stage ICR mouse embryos, which were cultured to produce blastocysts and then transferred to pseudopregnant ICR females. The resulting chimeric male mice were crossed with female C57BL/6N mice to establish the $Ox1r^{flox\text{-}neo/+}$ line. $Ox1r^{flox\text{-}neo/+}$ mice were crossed with *Actb-Flp* knock-in mice to remove the neomycin resistance gene with the Flp-FRT system. $Ox1r^{flox\text{-}neo/+}$ mice were further crossed with Ayu1-Cre mice to establish *Ox1r*-deficient mice (See Figure S1).

Mouse model of diet-induced obesity

C57BL/6 mice were fed a high-fat diet (D12492; Research Diet) starting after weaning at 3-4 weeks of age. The low-fat diet or normal chow (MF; Oriental Yeast) provided 3.6 kcal/g (61% carbohydrate, 26% protein, and 13% fat), whereas the high-fat diet provided 5.2 kcal/g (20% carbohydrate, 20% protein, and 60% fat). After 9 weeks of being fed the high-fat diet, mice were sacrificed at ZT11. Some cages were equipped with a running wheel (#ENV-044, Med

Associates).

Measurement of daily food intake

The mice were individually housed and fed powdered chow (MF; Oriental Yeast) or high-fat diet (D12492; Research Diet) in a small food jar. After one week of habituation to the powdered food, the amount of food intake was measured every 24 hours for four consecutive days.

Physical parameters

Mice were extended to their full length by the same experimenter, and the snout-to-anus distance was determined to measure the body length. The BMI was calculated as weight in grams divided by the square of the height in centimeters.

Blood analysis

Blood glucose levels were measured in blood from the tail using Glutest kits (Sanwa Kagaku). Blood was collected from the tail vein or orbital sinus of anesthetized mice to measure insulin and leptin levels. The blood was centrifuged, and then the serum was stored at -80ºC until use. Samples were analyzed using Mouse Insulin ELISA kits and Mouse Leptin ELISA kits (Morinaga Institute of Biological Science, Yokohama, Japan).

Indirect calorimetry

Indirect calorimetry was performed using an ARCO system (ARCO, Japan). For genetic studies, 10- to 12-week-old animals were individually housed in calorimeter chambers for more than 3 days for acclimatization and then data were collected for 5-6 days. The mice were fed chow or a high-fat diet from the age of 3 weeks. Raw energy expenditure was calculated as (3.815 + 1:2323 x respiratory quotient (RQ) x oxygen consumption. RQ was calculated as the ratio of carbon dioxide production to oxygen consumption.

Tissue preparation

Mice were sacrificed via cervical dislocation under deep anesthesia with sodium pentobarbital (50 mg/kg body weight). Then, the brain was rapidly removed and the medial hypothalamus was dissected on ice based on the following boundaries: rostral, the optic chiasm; caudal, the mammillary bodies; 1 mm bilateral from the midline; and 1.5 mm dorsal of the ventral surface. This dissected tissue included the arcuate nucleus, ventromedial hypothalamic nucleus,

dorsomedial hypothalamic nucleus, paraventricular hypothalamic nucleus, anterior hypothalamic area, and the medial half of the lateral hypothalamic area.

Quantitative RT-PCR

Total RNA was isolated using the RNeasy Lipid Tissue Mini kit (Qiagen, Chatsworth, CA) and used for cDNA synthesis with oligo dT primers and a PrimeScript reverse transcriptase kit (TaKaRa). Real-time quantitative PCR reactions were performed with the ViiA7 Real-Time PCR System (Thermo Fisher) using SYBR GREEN PreMix Ex Taq (TaKaRa). The following PCR primers were used: *Agrp* forward, 5 -TCCCAGAGTTCCCAGGTCTA-3; *Agrp* reverse, 5 - GCCAAAGCTTCTGCCTTCT-3; *Avp* forward, 5 -AGGATGCTCAACACTACGCTCT -3; *Avp* reverse, 5 -ACTGTCTCAGCTCCATGTCAGA -3; *Crh* forward, 5 - GAAAGGGAAAAGGCAAAAGAA-3; *Crh* reverse, 5 -GTTAGGGGCGCTCTCTTCTC-3; *Gapdh* forward, 5 -AGAACATCATCCCTGCATCC-3; *Gapdh* reverse, 5 - CACATTGGGGGTAGGAACAC-3; *Ghrh* forward, 5 -CTCTTTGTGATCCTCATCCTCAC-3; *Ghrh* reverse, 5 -AGTTTCCTGTAGTTGGTGGTGAA-3; *Mc4r* forward, 5 - GCCAGGGTACCAACATGAAG-3; *Mc4r* reverse, 5 -ATGAAGCACACGCAGTATGG-3; *Mch* forward, 5 -TGCTGAGTCCACACAGGAAA-3; *Mch* reverse, 5 -GCCAACATGGTCGGTAGACT-3; *Npy* forward, 5 -TACTCCGCTCTGCGACACTA-3; *Npy* reverse, 5 - TCACCACATGGAAGGGTCTT-3; *Orexin* forward, 5 -GGGTATTTGGACCACTGCAC-3; *Orexin* reverse, 5 -CCCAGGGAACCTTTGTAGAAG-3; *Oxytocin* forward, 5 - GCCAGGAGGAGAACTACCTG-3; *Oxytocin* reverse, 5 -CTCCGAGAAGGCAGACTCAG-3; *Pacap* forward, 5 -CTATGGCTATTGCTATGCACTCTG-3; *Pacap* reverse, 5 - CAACCTGGGGAAGACTCATTTAG-3; *Pomc* forward, 5 -AACCTGCTGGCTTGCATC-3; Pomc reverse, 5 -TTTTCAGTCAGGGGCTGTTC-3; *Sim1* forward, 5 -CCTCCATCCACAGAATCCAC-3; *Sim1* reverse, 5 -TGATACTGTTCGGTGCGGTA-3; *Somatostatin* forward, 5 - CTCTGCATCGTCCTGGCTTT-3; *Somatostatin* reverse, 5 -AAGTACTTGGCCAGTTCCTGTTT-3; *Trh* forward, 5 -GAAGGTGCTGTGACTCCTGAC-3; *Trh* reverse, 5 - ATCTAAGGCAGCACCAAGGTC-3;

A relative quantification method was employed to quantify the expression of the target mRNAs by calculating the ratio between the amount of the target mRNA and a reference mRNA within the same sample, according to the manufacturer’s protocol. The reactions were performed in duplicate and the results were averaged. The average levels of the *glyceraldehyde-3-phosphate dehydrogenase (GAPDH)* mRNA were used for normalization.

Body weight gain of mice fed a high-fat diet in a cage equipped with a running wheel

Male and female wild-type and orexin neuron-ablated mice were randomly assigned to either chow or a high-fat diet (D12451; Research Diet) and housed in a cage equipped with or without a running wheel (ENF-044; Med Associates). Mice were group-housed after the ages of 6 weeks. Body weight was measured weekly from the ages of 4 weeks to 15 weeks.

Histological assessment of brown adipose tissue (BAT)

Mice were deeply anesthetized with isoflurane and transcardially perfused with phosphate-buffered saline (PBS) followed by 4% paraformaldehyde in PBS. The BAT was postfixed with 4% PFA at 4°C overnight. The fixed tissues were embedded in paraffin. The BAT was sectioned at a thickness of 8 μm and processed for hematoxylin-eosin staining. Images of the stained sections were acquired using LSM700 with Plan-Apochromat 20x/0.8 M27 (Zeiss). Images were converted to 8-bit grayscale and then thresholded with a size filter using ImageJ software (National Institutes of Health) to determine the lipid droplet area (See Figure S2). We delineated the cell borders of all adipocytes in each image (450 μm x 350 μm) with a dark pen and then counted the number of adipocytes. The adipocyte number was recalculated as the number per square millimeter. The histological assessment was performed by an investigator who was blinded to the animal information.

Immunoblotting

A rabbit monoclonal antibody against UCP1 (clone D9D6X, #14670, Cell Signaling Technologies) was used as a primary antibody. Mice were sacrificed via cervical dislocation under deep anesthesia with sodium pentobarbital (50 mg/kg body weight). Then, the BAT was rapidly removed, frozen in liquid nitrogen and stored at -80°C until use. The BAT was homogenized using a rotor-stator homogenizer (Polytron) in ice-cold lysis buffer (20 mM HEPES pH7.5, 100 mM NaCl, 10 mM Na4P2O7, 1.5% Triton-X100,15 mM NaF, 1X PhosSTOP (Roche), 5 mM EDTA, 1X Protease Inhibitor (Roche)), and then centrifuged at 13,000g at 4 °C. The supernatants were separated by SDS–PAGE and transferred on PVDF membrane. Blots were subsequently washed in PBS plus 0.1 % Tween-20 (PBS-T) and incubated overnight at -4°C with primary antibody (1:1000) in PBS-T with 5% bovine serum albumin. The blot was then washed and incubated with horseradish peroxidase-conjugated, donkey anti-rabbit IgG (Jackson ImmunoReserarch; 1:5,000 dilution in 5% skim milk and PBS-T). After washed, the blot was

exposed to Clarity Western ECL Substrate (Bio-Rad). The chemiluminescence signaling was detected using FUSION Solo 6S.EDGE (Vilber-Lourmat).

Statistics

Sample sizes were determined using R software based on averages and standard deviations that were obtained from small-scale experiments or from our previous data (Funato et al., 2009). No method of randomization was used in any of the experiments. The experimenters were blinded to genotypes and treatment assignments. Statistical analyses were performed using SPSS Statistics 22 software (IBM). We first performed a two-way ANOVA using diet and genotype as the two independent variables to assess the significance of differences in most parameters, such as body weights and leptin levels. When a significant interaction between diet and genotype was found, we performed an ANOVA on the results from each diet group followed by Tukey's test. When the two-way ANOVA detected significant effect of the genotype, we further performed a Tukey's test of the genotype. We performed two-tailed Students T-test with Bonferroni correction to compare parameters from mice of the same genotype between regular chow and a high-fat diet. $P < 0.05$ was considered statistically significant. All data are presented as mean ± SEM.
